# Circular RNAs are Potential Prognostic Markers of Head and Neck Squamous Cell Carcinoma: Findings of a Meta-Analysis Study

**DOI:** 10.3389/fonc.2022.782439

**Published:** 2022-02-28

**Authors:** Moumita Nath, Dibakar Roy, Yashmin Choudhury

**Affiliations:** Department of Biotechnology, Assam University, Silchar, India

**Keywords:** circular RNA, HNSCC, meta-analysis, overall survival, prognostic markers

## Abstract

**Background:**

Several studies have reported the role of circRNAs in the pathogenesis, diagnosis and prognosis of different cancers. This meta-analysis study aimed to evaluate the potential of using circRNAs as prognostic biomarkers of head and neck squamous cell carcinoma (HNSCC).

**Methods:**

816 relevant articles were retrieved from PubMed and Science Direct databases, out of which 17 met the inclusion criteria. These 17 studies were assessed for quality by the Newcastle-Ottawa Scale (NOS) system, and 9 high quality studies (NOS>7) were included in the meta-analysis. Cochran Q test and the I square (*I*^2)^ metric were calculated to detect potential heterogeneity among studies. Sensitivity analysis was performed to validate the credibility of outcomes, and publication bias was determined using Begg’s funnel plot and Egger’s test. Hazard ratio (HR) and 95% Confidence Intervals (CIs) were used to evaluate overall survival (OS) of HNSCC patients by univariate and multivariate analyses.

**Results:**

The dysregulated levels of 9 circRNAs (circPVT1, circCORO1C, circ_0000199, circCUX1, circPARD3, circMYC, circ_0102272, circ_0092125 and circ_00072387) were inversely related to OS of HNSCC patients [upregulated circRNA (univariate analysis: HR = 3.40, 95% CI: 2.66-4.36, p < 0.0001, *I*^2^ = 0%; multivariate analysis: HR = 3.33, 95% CI: 2.54-4.38, p < 0.0001, *I*^2^ = 0%), downregulated circRNA (univariate analysis: HR = 2.83, 95% CI: 1.73-4.65, p < 0.0001, *I^2^* = 57.8%; multivariate analysis: HR = 2.35, 95% CI: 1.42-3.89, p = 0.0009, *I^2^* = 0%)]. The individual HR for these 9 circRNAs indicated inverse relation to OS, validating the overall HR. The dyregulated levels of these circRNAs were also associated with poor clinicopathological outcomes such as primary tumor size, lymph node metastasis, distant metastasis and poor tumor (T), nodes (N), metastases (M); i.e TNM staging, and six of these circRNAs regulated diverse micro RNAs, revealing their role in tumorigenesis and cancer progression.

**Conclusion:**

Nine different circRNAs dysregulated in HNSCC tumors may serve as potential prognostic markers of HNSCC. These markers are associated with reduced OS and poor clinicopathological outcomes of HNSCC patients. They are also involved in the pathogenesis and progression of HNSCC through diverse mechanisms.

## Introduction

Head and neck cancers are a group of aggressive and genetically complex cancers arising from diverse anatomical sites of the head and neck (1). According to the GLOBOCAN 2020 database, out of an estimated 19.3 million new cancer cases, there were a cumulative number of 878348 (4.5%) new cases of cancer of the lip and oral cavity, larynx, nasopharynx, orpharynx, and hypopharynx reported in 2020, worldwide (2). The incidence rate of lip and oral cavity cancer (Age Standardized Rate 10.2 per 100,000) follows that of prostate and lung cancer as the leading cancers among males in the lower Human Development Index (HDI) countries, and is the leading cause of cancer-related death among males in India. The high incidence of cancers of the lip and oral cavity cancers in South Central Asia and Melanesia (Papua New Guinea) is largely attributed to the popularity of betel nut chewing in these regions. On the other hand, the high incidence rates in Eastern and Western Europe and in Australia/New Zealand have been attributed to alcohol consumption, tobacco smoking and HPV infection for cancers of the oropharyngeal region, and to ultraviolet radiation from sunlight exposure for lip cancer (2). While most of these cancers, collectively known as head and neck squamous cell carcinoma (HNSCC) are derived from the mucosal epithelium of the oral cavity, pharynx and larynx (1, 3), their heterogeneity at the molecular level has proved to be a major impediment in the identification of drug targets and targeted therapeutics (1). Thus, the 5-year overall survival (OS) rate of HNSCCs has remained approximately at 40–50% without much improvement over the past decade, largely due to poor availability of effective therapeutic options for HNSCC patients with recurrent disease (4). Additionally, locoregional recurrences occur in 15%-50% of patients with HNSCC and is a major factor contributing towards deaths because of the complexities associated with treatment of recurrent HNSCC, which include the effects of prior treatment on tumor cells and the infiltrative and the multifocal nature of HNSCC itself (5).

Majority of HNSCC patients present with advanced stage cancer (3) which is difficult to treat even using a combinatorial approach (1), thereby underscoring the significance of early detection. Careful physical examination and early recognition of oral pre-malignant lesions (OPLs) which present as leukoplakia (white patches) or erythroplakia (red patches), and may progress to invasive cancer, remain the cornerstone of the early detection of HNSCC (3). Indeed, identification of early, asymptomatic oral squamous cell carcinoma (OSCC) during routine oral examinations by community-based dentists and physicians followed by oral brush biopsy could increase the 5 year OS rate to a remarkable 94% (6). Prognosis of the cancer and determination of the likelihood of disease recurrence or progression is equally important. Thus, the recent years have seen the development of significant interest in the formulation of strategies based on specific markers and molecular signatures for early detection and/or clinical prognosis of HNSCC (4, 7), with non-invasive diagnostic testing holding particular appeal (7). Key amongst these are “liquid biopsies,” which involve analysis of body fluid samples such as blood or other accessible fluids like ascites, pleural effusions, saliva, or urine. Most liquid biopsy-based diagnostic tests for solid malignancies are based on blood, serum or plasma specimens, which are less invasive, and have the potential to detect HNSCC, tumor recurrences or metastases at initial stages (7, 8). The primary markers being investigated from liquid biopsies are circulating tumor DNA (ctDNA) and circulating tumor cells (CTCs), tumor antigens, cell free coding and noncoding RNAs, metabolites as well as extracellular vesicles and exosomes (7, 8).

High throughput transcriptome analysis techniques have recently revealed circular RNAs (circRNAs) as potentially useful markers for the prognosis of cancer from body fluids. CircRNAs, also known as competitive endogenous RNAs (ceRNAs), are a large family of covalently closed, single-stranded, stable RNA molecules which regulate gene expression by regulating micro RNAs (miRNAs), binding with RNA-binding proteins (RBPs) or by getting directly translated into proteins. They exist stably and at high levels in body fluids, including plasma, serum, exosomes, and urine, and exhibit distinct expression patterns between patients with cancer and healthy controls (9, 10). Different circRNAs have been identified as functional prognostic biomarkers in lung, breast, colorectal, prostate, gastric, cervical, thyroid, bladder and ovarian cancers, hepatocellular carcinoma and esophageal squamous cell carcinoma (ESCC) (9, 11, 12). The circRNAs were found to be either significantly upregulated or downregulated in the body fluids of cancer patients in comparison to healthy controls with high discriminatory accuracy, and the expression level of most circRNAs were associated with tumour size, tumour differentiation, distant metastasis, and TNM stage (9).

The circular RNA, circPVT1, was proposed to play a pivotal and oncogenic role in the pathogenesis of HNSCC. CircPVT1 was found to be over-expressed in tumors compared to matched non-tumoral tissues, with particular enrichment in patients with TP53 mutations, and circPVT1 upregulation and downregulation were respectively associated with an increase or decrease in the malignant phenotype of HNSCC cell lines (13), indicating its relevance as a prognostic marker. Dysregulated circRNA expression has been reported in different types of HNSCC like ESCC, OSCC, laryngeal squamous cell carcinoma (LSCC), nasopharyngeal cancer and hypopharyngeal cancer (14–18).

In addition to the high heterogeneity of the molecular factors associated with HNSCC, inconsistencies are likely to arise among the findings of different studies on the potential of using circRNA as a biomarker of HNSCC due to variation in population ethnicity, sample size, specimens and control sources, which can become a hurdle to the application of circRNA in clinical practice (19). Therefore, we have performed a meta-analysis in order to investigate the relationship between the dysregulated expression of circRNAs and the prognosis and clinicopathological characteristics of HNSCC with the aim of determining whether circRNAs may be candidate biomarkers for HNSCC prognosis.

## Methods

### Literature Search Strategy

This study was conducted in accordance with the Preferred Reporting Items for Systematic Reviews and Meta-Analyses (PRISMA) checklist (20). The studies were retrieved from PubMed, and ScienceDirect databases using the search terms “Circular RNA AND Head & Neck Cancer”, “circRNA in Head and Neck Cancer”, “Circular RNA AND Head & Neck Cancer biomarker”, “Circular RNA AND HNSCC”, “circRNA AND HNSCC”, “circRNA AND prognosis of HNSCC”. Studies published from 20^th^ December 2017 to 10^th^ September 2021 were included. The search was advanced using the term ‘AND’ to recover the desired outcomes. The potentially significant records were recovered from this search by all authors by surveying the title and abstract of each article, which were additionally inspected cautiously for meta-analysis eligibility. Furthermore, the reference lists of the retrieved articles were searched physically in order to recover more qualified studies for the meta-analysis. In addition to the articles screened for eligibility in meta-analysis, articles relevant for writing the Introduction, Methods and Discussion sections of this study were also included from the above mentioned and other sources.

### Eligibility Criteria

The following inclusion criteria were predetermined for selecting articles eligible for meta-analysis (1) original research articles that reported data on the involvement of aberrantly expressed circRNAs in HNSCC patients; (2) articles reporting the link between circRNAs and survival outcomes in HNSCC patients; (3) studies on pathologically confirmed HNSCC (4) articles reporting the prognostic ability of circRNAs in HNSCC with available HR values and 95% confidence interval (CI); (5) studies that investigated the association between circRNAs and the clinicopathological features of HNSCC. The criteria for exclusion of articles were (1) articles not published in English (2) articles on the role of circRNAs in cancers other than HNSCC (3) reviews, basic studies, comments, meta-analyses, letters or case reports (4) low quality studies and (5) articles reporting the application of circRNAs in HNSCC diagnosis.

### Data Extraction and Quality Assessment

All data was extracted from the included articles by two investigators (DR and MN) carefully and independently in order to retain accuracy of the data. The information collected from each study were: (1) basic information including author’s name, year of publication, number of cases, number of controls (2) clinicopathologic information such as circRNAs profiles, altered expression, specimen type, detection method and TNM stage (3) prognostic data including the univariate and multivariate HR values with 95% CI for OS.

Eligible studies retrieved after applying the inclusion and exclusion criteria were further screened for quality by the NOS (Newcastle-Ottawa Scale) system (**Table 1**). The NOS system is a semi-quantitative system for determining the quality of studies and comprises of three dimensions *viz;* selection, comparability, and study type, which are further subdivided into eight categories. The NOS ranges between 0 and 9 score, with NOS > 7 indicating high quality studies (35). Thus, only studies with NOS > 7 were finally selected for inclusion in meta-analysis.

**Table 1:** Quality assessment of prognostic studies included on basis of the Newcastle-Ottawa Scale (NOS).

Study	Representativeness of the exposed cohorts	Selection of the non-exposed cohorts	Ascertainment of exposure	Demonstration that outcome of interest was not present at start of study	Comparability of cohorts on the basis of the design or analysis	Assessment of outcome	Was follow-up long enough for outcomes to occur?	Adequacy of follow up of cohorts	Total NOS score	Status
Verduci et al. (13) (circPVT1)	1	1	1	1	1	1	1	1	8	Included
Wu et al. (21) (circCORO1C)	1	1	1	1	1	1	1	1	8	Included
Gao et al. (22) (circPARD3)	1	1	1	1	1	1	1	1	8	Included
Li et al. (23) (circTGFBR2)	1	1	1	1	1	1	0	0	6	Not Included
Luo et al. (24) (circ_0000199)	1	1	1	1	1	1	1	1	8	Included
Liu et al. (25) (circRPMS1)	1	1	1	1	1	0	0	0	5	Not Included
Feng et al. (26) (circ_0008287 & circ_0005027)	1	0	1	0	0	0	0	0	2	Not Included
Wang et al. (27) (circ_036186)	1	0	1	0	1	0	0	0	3	Not Included
Wang et al. (28) (circMATR3)	1	1	1	1	1	0	1	0	6	Not Included
Shuai et al. (29) (circ_0000285)	1	1	1	1	1	0	0	0	5	Not Included
Wu et al. (18)(circCUX1)	1	1	1	1	1	1	1	1	8	Included
Tian et al. (30) (circRASSF2)	1	1	1	0	1	0	0	0	4	Not Included
Wei et al. (31) (circ_0042666)	1	0	1	1	1	0	0	0	4	Not Included
Luo et al. (42) (circMYC)	1	1	1	1	1	1	1	1	8	Included
Liu et al. (32)(circ_0102272)	1	1	1	1	1	1	1	1	8	Included
Gao et al. (33) (circ_0092125)	1	1	1	1	1	1	1	1	8	Included
Dou et al. (34) (circ_00072387)	1	1	1	1	1	1	1	1	8	Included

### Statistical Analysis

Hazard ratio (HR) and 95% CIs were used to evaluate overall survival (OS) of HNSCC patients. Heterogeneity among the included studies for meta-analysis were examined by Cochran’s Q test (36), with P < 0.05 considered to be significant. The *I^2^* statistic (37) was used to evaluate the percentage of the total variation due to heterogeneity, such that *I^2^* values of 0% - 25% suggest no heterogeneity, while values of 25% - 50%, 50% - 75% and 75% - 100% indicate reasonable heterogeneity, huge heterogeneity and outrageous heterogeneity, respectively. In the presence of significant heterogeneity (Q statistic P < 0.05), the random effect model (38) was used to calculate the pooled HR, else the fixed effect model (39) was used. Sensitivity analysis was performed in order to validate the credibility of outcomes by chronological omission of individual studies. All investigations were performed by utilizing the StatsDirect statistical software (Version 2.7.2). Publication bias was determined using Begg’s funnel plot and Egger’s test (40, 41).

## Results

### Search Results

**Figure 1:**
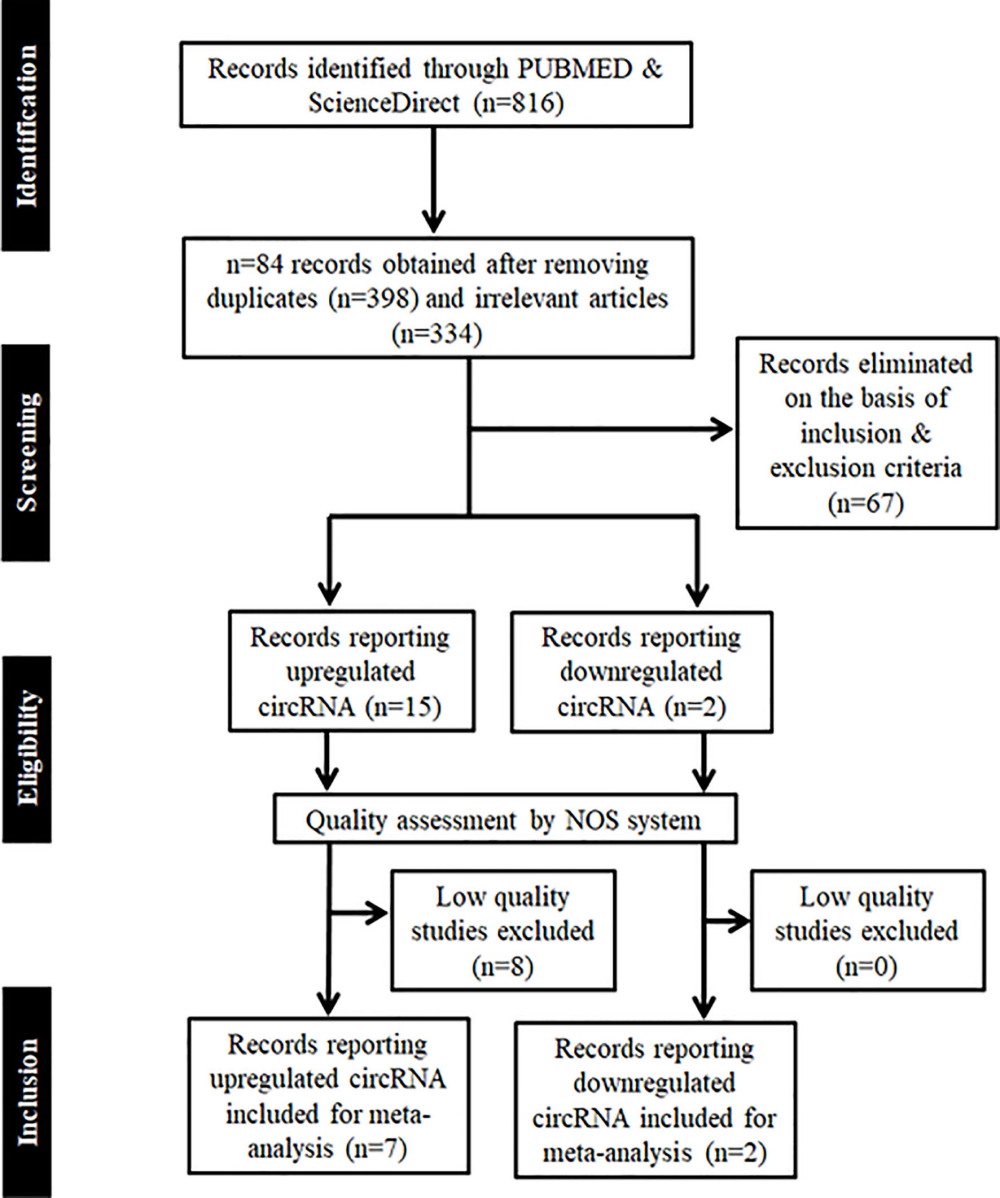
PRISMA flow chart showing the methodology employed for literature retrieval for meta-analysis. Records were screened on the basis of inclusion and exclusion criteria, whose quality was further assessed by NOS system.

The methodology employed for literature search is shown in **Figure 1**. After a thorough search of PubMed and ScienceDirect databases using the mentioned search terms and a manual search through reference lists, a total of 816 records which appeared to be relevant to the role of circRNAs in HNSCC were retrieved. 84 articles were found to be relevant after reading the article titles and abstracts and removal of the duplicate articles (n=398) and irrelevant articles (n=334). These 84 articles were further screened on the basis of the inclusion and exclusion criteria to determine their eligibility for meta-analysis, and 67 articles were eliminated. The remaining 17 articles were further segregated as records reporting upregulated circRNAs and downregulated circRNAs in HNSCC, respectively. These 17 records were screened for quality by the NOS system, leading to the exclusion of 8 studies due to a low NOS score (NOS < 7) (**Table 1**). Finally, 7 high quality studies reporting upregulated circRNAs and 2 studies reporting downregulated circRNAs were included in the quantitative meta-analysis.

In addition, 8 articles were included as references to the methods used for meta-analysis and 25 articles were included for writing the introduction and discussion sections of this article.

### Characteristics of Included Studies

The NOS was used to evaluate the quality of the included studies and the nine included studies received high scores (NOS>7) (**Table 1**) indicating a high quality of these studies, and ensuring the reliability of the overall result. The baseline data from the 9 included studies is shown in **Table 2**. Collectively, these studies comprised a total number of 982 patients who were pathologically confirmed as suffering from HNSCC. These circRNAs were detected by the techniques of RNA Sequencing (18) or quantitative polymerase chain reaction, qPCR (13, 21, 22, 24, 32–34, 42). All of these circular RNAs were dysregulated in cancer tissue and were associated with poor clinicopathological outcomes such as primary tumor size, lymph node metastasis, distant metastasis and tumor (T), nodes (N), metastases (M); i.e TNM stage. Upregulated levels of circCUX1 in hypopharyngeal cancer tissue in comparison to adjacent normal tissue correlated significantly with primary tumor size, lymph node metastasis, distant metastasis and TNM stage (18). High circPARD3 levels in LSCC tissue in comparison to adjacent normal mucosa positively correlated with T stage, N stage and clinical stages, and increased progressively from T1 to T4 (22). Similarly, OSCC patients with higher exosomal circ_0000199 had higher tumor recurrence rate and higher mortality than patients with lower levels of exosomal circ_0000199 (24). Wu et al., reported that circCORO1C expression was relatively abundant in LSCC than in adjacent normal mucosa, and was significantly correlated with T stage, N stage and clinical stage (21). In thyroid cancer patients, high levels of circ_0102272 was associated with lymph node metastasis along with advanced clinical stage indicating shorter overall survival in comparison to patients with low expression of circ_0102272 (32). Exosomal circMYC acts as an independent predictor of nasopharyngeal carcinoma patient survival as its high expression is closely associated with tumor size, lymph node metastasis and TNM stage (42). Apart from upregulated circRNAs, the downregulation of two circRNAs (circ_0092125, circ_0072387) were significantly correlated with tumor size, TNM stage, and lymph node metastasis in OSCC patients (33, 34). Kaplan- Meir analysis also revealed a correlation between the upregulation of these seven circRNAs (circPVT1, circCOROC1, circPARD3, circ_0000199, circ_0102272, circMYC and circCUX1) (13, 18, 21, 22, 24, 32, 42) and downregulation of two circRNAs (circ_0092125, circ_0072387) (33, 34) with inferior OS of patients of HNSCC patients.

**Table 2:** Characteristics of the included studies.

Study	Patient number	Control type	Sample type	Case size		CircRNA signature	TNM Stage (I, II, III, IV)	Expression status	Method	Survival indicator	Follow-up time	References
				High	Low							
Luo et al., 2020	108	Paired healthy counterparts	Serum	40	68	circ_0000199	**High**: I or II:33; III or IV:35 **Low**: I or II:28; III or IV:12	Upregulated	qRT-PCR	OS	Unclear	(24)
Luo et al., 2020	210	Paired healthy counterparts	Serum	148	62	circMYC	**High:** I or II:76; III or IV:72 **Low:** I or II:48; III or IV:14	Upregulated	qRT-PCR	OS	Unclear	(42)
Liu et al., 2020	58	Adjacent normal tissue	Tissue	33	25	Circ0102272	**High:** I-II:19; III-IV:14 **Low:** I-II:21; III-IV:4	Upregulated	qRT-PCR	OS	Unclear	(32)
Wu et al., 2021	78	Adjacent normal tissue	Tissue	45	33	circCUX1	**High**: I or II:15; III or IV:30 **Low**: I or II:20; III or IV:13	Upregulated	RNA sequencing	OS	Unclear	(18)
Verduci et al., 2017	115	Histologically normal tissue	Tissue	67	48	circPVT1	Unclear	Upregulated	qRT-PCR	OS	≥ 12 months	(13)
Gao et al., 2020	100	Adjacent normal mucosa tissue	Tissue	30	70	circPARD3	I+II=48; III+IV=52	Upregulated	qRT-PCR	OS	Unclear	(22)
Wu et al., 2020	164	Adjacent normal mucosa tissue	Tissue	57	107	circCORO1C	**High**:I+II=55 **Low :** III+IV=52	Upregulated	qRT-PCR	OS	Unclear	(21)
Gao et al., 2019	86	Adjacent normal tissue	Tissue	50	36	circ0092125	**High:** I:22 II:18 III:5 IV:5 **Low:** I:7 II:14 III:6 IV:9	Downregulated	qRT-PCR	OS	Unclear	(33)
Dou et al., 2019	63	Adjacent normal tissue	Tissue	NS	NS	circ00072387	I:14 II+III:39 IV:10	Downregulated	qRT-PCR	OS	Unclear	(34)

*NS, Not specified.

### Meta-Analysis for Overall Survival

**Figure 2:**
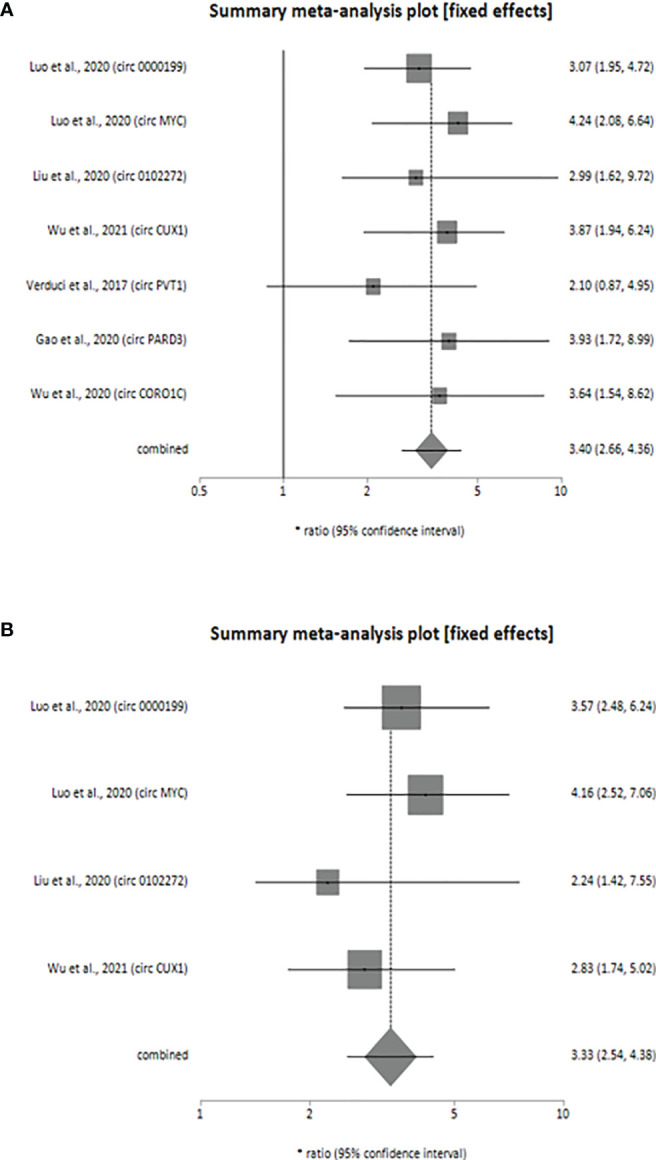
Forest plot showing the combined HR along with individual HR values at 95% CI for the upregulated circRNA profiles in predicting the overall survival of patients with HNSCC **(A)** univariate analysis, **(B)** multivariate analysis. Square shapes indicate the effect size of each upregulated circRNA profile, while diamond shapes indicate the overall effect size. The 95% CI of each effect sizes are indicated by respective bars.

Our meta-analysis indicates that the upregulated levels of 7 circRNAs (circ_0000199, circ MYC, circ_0102272, circCUX1, circPVT1, circPARD3 and circCORO1C) were inversely related to OS of HNSCC patients (univariate analysis: HR = 3.40, 95% CI: 2.66-4.36, p < 0.0001, *I*^2^ = 0%; multivariate analysis: HR = 3.33, 95% CI: 2.54-4.38, p < 0.0001, *I*^2^ = 0%). Also, the individual HR for these 7 circRNAs [circ_0000199 (univariate analysis: HR=3.07, 95% CI: 1.95-4.72; multivariate analysis: HR=3.57, 95% CI: 2.48-6.24), circ MYC (univariate analysis: HR=4.24, 95% CI: 2.08-6.64; multivariate analysis: HR=4.16, 95% CI: 2.52-7.06), circ 0102272 (univariate analysis: HR=2.99, 95% CI: 1.62-9.72; multivariate analysis: HR=2.24, 95% CI: 1.42-7.55), circCUX1 (univariate analysis: HR=3.87, 95% CI: 1.94-6.24; multivariate analysis: HR=2.83, 95% CI: 1.74-5.02), circPVT1 (univariate analysis: HR=2.10, 95% CI: 0.87-4.95), circPARD3 (HR=3.93, 95% CI:1.72-8.99), circCORO1C (HR=3.64, 95% CI: 1.54-8.62), indicated inverse relation to OS of HNSCC patients validating the overall HR calculated for both univariate (**Figure 2A**) and multivariate (**Figure 2B**) analyses. The variables used for multivariate analyses were areca nut use (24), TNM stage (18, 24, 42), lymphatic metastasis (18, 24, 32, 42), tumor size (18, 24, 42) and distant metastasis (18). Finally, the statistical results show that the various circRNAs had no significant heterogeneity (*I^2^* = 0%, p > 0.05) and thus, a fixed-effect model was used for both the analyses.

**Figure 3:**
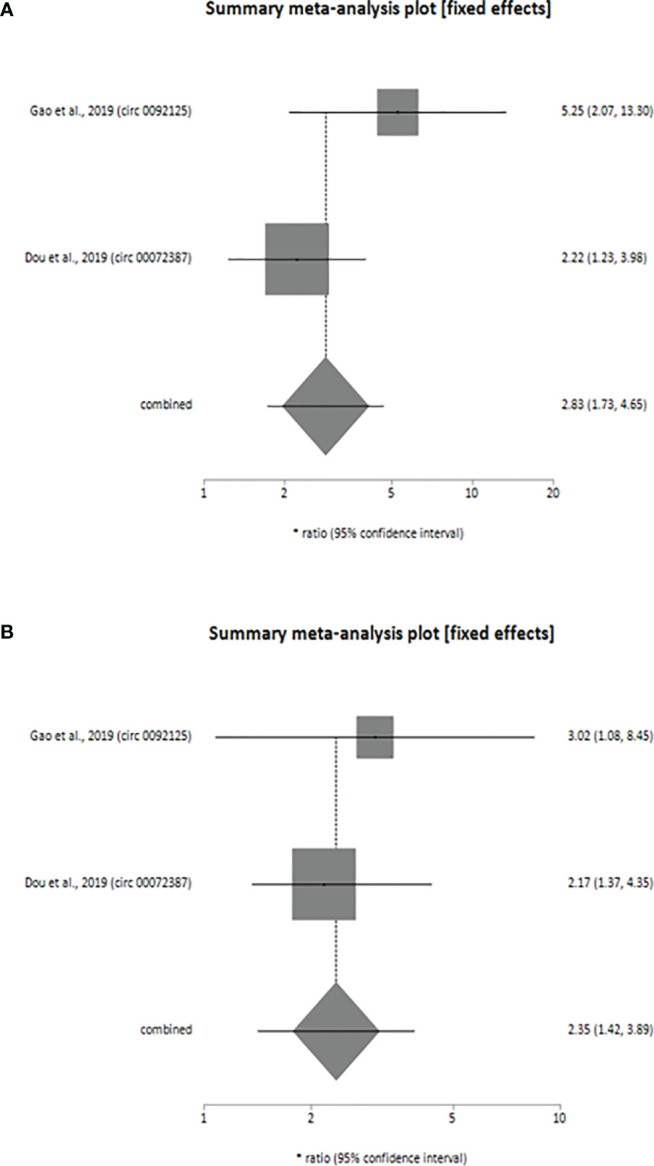
Forest plot showing the combined HR along with individual HR values at 95% CI for the downregulated circRNA profiles in predicting the overall survival of patients with HNSCC **(A)** univariate analysis, **(B)** multivariate analysis. Square shapes indicate the effect size of each upregulated circRNA profile, while diamond shapes indicate the overall effect size. The 95% CI of each effect sizes are indicated by respective bars.

Additionally, downregulation of 2 circRNAs (circ_0092125 and circ_00072387) was associated with poor prognosis of HNSCC patients as revealed by our meta-analysis (univariate analysis: HR=2.83, 95% CI: 1.73-4.65, p < 0.0001, *I^2^* = 57.8%; multivariate analysis: HR=2.35, 95% CI: 1.42-3.89, p < 0.0009, *I^2^* = 0%). The variables used for multivariate analyses were TNM stage (33, 34), tumor size (33) and lymph node metastasis (33). Like upregulated circRNAs, the individual HR for these 2 circRNAs [circ_0092125 (univariate analysis: HR=5.25, 95% CI: 2.07-13.30; multivariate analysis: HR=3.02, 95% CI: 1.08-8.45), circ_00072387 (univariate analysis: HR=2.22, 95% CI: 1.23-3.98; multivariate analysis: HR=2.17, 95% CI: 1.37-4.35) also indicated poor prognosis of HNSCC patients, validating the overall HR calculated for both univariate (**Figure 3A**) and multivariate (**Figure 3B**) analyses. The statistical results show that the circRNAs had no significant heterogeneity (univariate analysis: *I^2^* = 57.8% p > 0.05, multivariate analysis: *I^2^* = 0%, p > 0.05) and thus, a fixed-effect model was used for both the analyses.

### Sensitivity Analysis and Publication Bias

**Figure 4:**
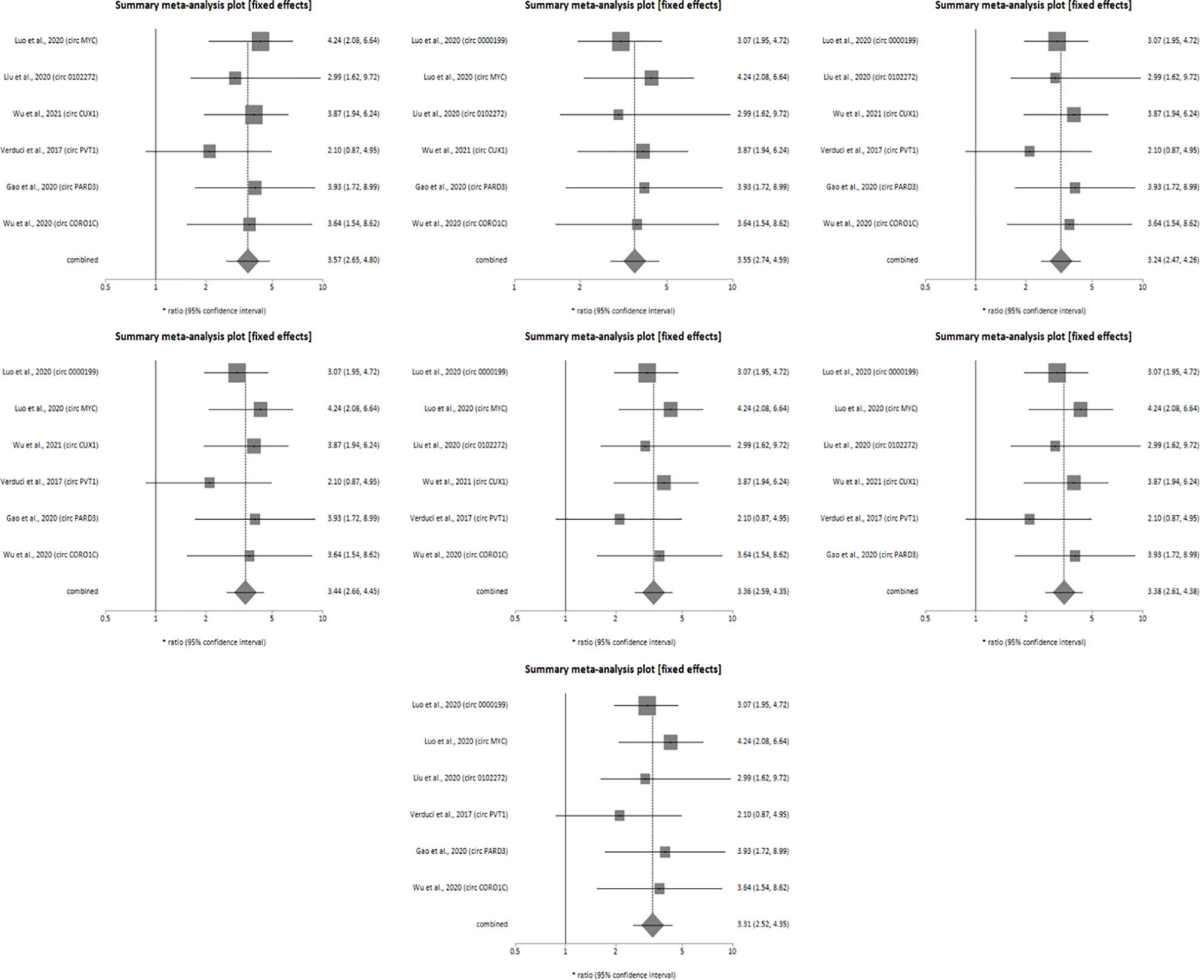
Forest plots of sensitivity analysis showing the combined HR values at 95% CI upon sequential deletion of the studies included in univariate analysis which reported upregulated circRNAs. Square shapes indicate the effect size of each upregulated circRNA profile, while diamond shapes indicate the overall effect size. The 95% CI of each effect sizes are indicated by respective bars.

**Figure 5:**
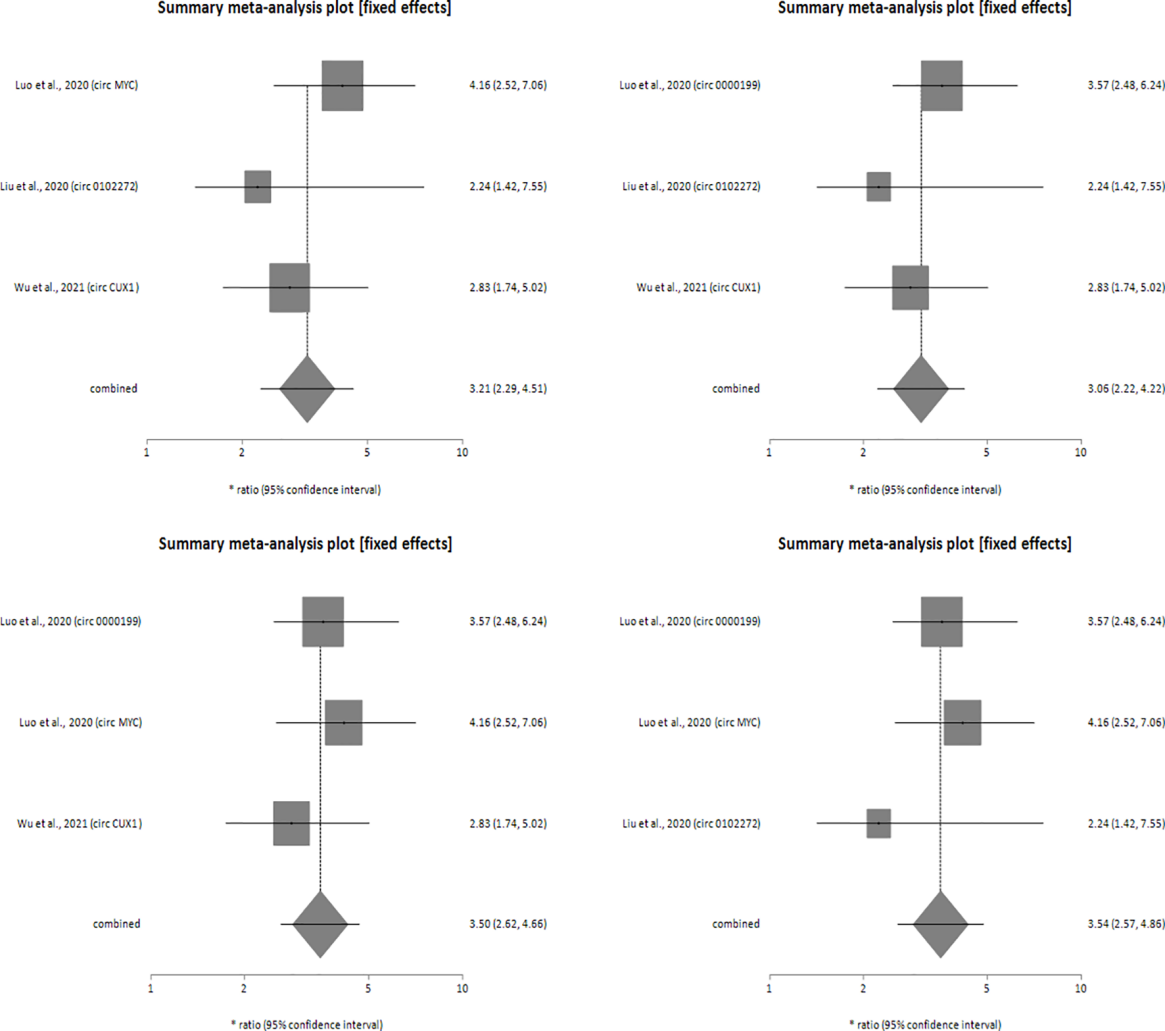
Forest plots of sensitivity analysis showing the combined HR values at 95% CI upon sequential deletion of the studies included in multivariate analysis which reported upregulated circRNAs. Square shapes indicate the effect size of each upregulated circRNA profile, while diamond shapes indicate the overall effect size. The 95% CI of each effect sizes are indicated by respective bars.

**Figure 6:**
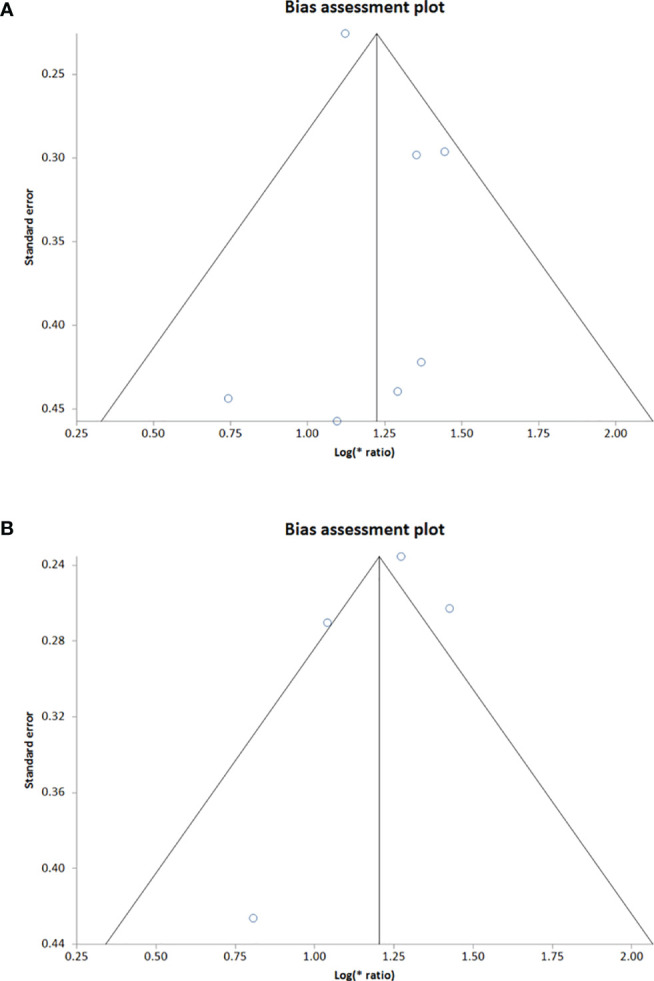
Begg’s funnel plot showing publication bias of upregulated **(A)** univariate analysis & **(B)** multivariate analysis. Each dot of the Begg’s funnel plot represents a separate study. The two border lines of the plot represent the pseudo 95% confidence intervals against their corresponding standard errors and the middle solid line indicates the overall effect from the meta-analysis.

Sensitivity analysis was performed for studies reporting upregulated level of circRNAs in HNSCC patients in order to determine the impact of individual studies on the pooled HR obtained by both univariate (**Figure 4**) and multivariate (**Figure 5**) analyses, and to validate whether any single study skewed the combined HR significantly. When the included studies of univariate analysis, namely Luo et al., 2020 (circ 0000199), Verduci et al., 2017 (circ PVT1), Luo et al., 2020 (circ MYC), Liu et al., 2020 (circ 0102272), Gao et al., 2020 (circ PARD3), Wu et al., 2020 (circ CORO1C) and Wu et al., 2021 (circ CUX1) were excluded one at a time, the combined HR values obtained (3.57, 3.55, 3.24, 3.44, 3.36, 3.38 and 3.31 respectively) did not indicate significant misrepresentation by any individual study in comparison to the overall HR value (HR = 3.40) (**Figure 4**). Similarly, sequential omission of included studies of multivariate analysis, namely, Luo et al., 2020 (circ 0000199), Luo et al., 2020 (circ MYC), Liu et al., 2020 (circ 0102272) and Wu et al., 2021 (circ CUX1) resulted in combined HR values (HR = 3.21, 3.06, 3.50 and 3.54 respectively) which did not skew the overall HR value (HR = 3.33) (**Figure 5**). Sensitivity analysis was not performed for meta-analysis conducted for downregulated circRNAs as only two studies met the inclusion criteria, and individual HR values of both the studies did not skew the combined HR (**Figures 3A, B**). Results based on Begg’s funnel plots show that there was no significant publication bias in both the analyses for upregulated circRNAs [univariate analysis (Begg’s test: p = 0.13, Egger’s test: p = 0.74), multivariate analysis (Begg’s test: p = 0.08, Egger’s test: p = 0.27) (**Figure 6**). However, Begg’s funnel plot and Egger’s test value could not be obtained for studies reporting downregulated circRNAs due to the small number of studies (the minimum number of studies required to generate a funnel plot is four).

## Discussion

The therapies for HNSCC currently available and administered alone or in combination are radiotherapy, chemotherapy, targeted drugs and immunotherapy. While radiotherapy and chemotherapy remain highly toxic, the efficacy of EGFR inhibitors which are the only approved targeted drugs, is limited by inherent and acquired resistance (1). While newer strategies have contributed towards a significant improvement of the quality of life in the past two decades (43), there is no improvement in OS rates of patients in recent decades. Thus, an improved understanding of the molecular mechanisms underlying HNSCC is necessary to enhance therapeutic efficacy. Besides this, the requirement to identify patients who are more susceptible to metastases and recurrences has led to a pressing need for more compelling biomarkers for the identification of individuals who are at higher risk. This indicates the critical requirement of novel biomarkers to more precisely predict outcome of HNSCCs and help in the decision of therapeutic strategies. Studies indicate that circRNA play a vital role in the regulation of various molecular events which determine the occurrence, progression, and metastasis of various malignant tumors (44). Studies have also demonstrated that circRNAs are relatively stable because of their covalently closed circular structures with neither 5’ caps nor 3’ tails; consequently, most circRNAs are resistant to RNA exonucleases, and are more stable when compared with linear transcripts (45), making them good candidates for biomarkers.

A number of meta-analysis studies have established circRNA as a prognostic biomarker for cancer (19), such as hepatocellular carcinoma (46), colorectal cancer (47) and breast cancer (48). However, to the best of our knowledge, no meta-analysis has been performed to determine the prognostic significance of circRNAs in HNSCCs. In performing this meta-analysis, we included 9 studies with a total of 982 HNSCC cases in order to uncover the connection between the expression of different types of circular RNA and their clinicopathological and prognostic significance in HNSCC. Our results indicate that in HNSCC patients, the high expressions of seven circRNAs (circPVT1, circCOROC1, circPARD3, circ_0000199, circMYC, circ_0102272 and circCUX1) and low expressions of two circRNAs (circ0092125 and circ00072387) in tumors, in comparison to the levels in adjacent normal tissue were associated with poor clinicopathological features such as poor TNM staging, histological grading, lymph node metastasis, and recurrence in HNSCC and showed inferior OS [increased circRNA expression (univariate analysis: HR = 3.40, 95% CI: 2.66-4.36, p < 0.0001, *I*^2^ = 0%; multivariate analysis: HR = 3.33, 95% CI: 2.54-4.38, p < 0.0001, *I*^2^ = 0%), decreased circRNA expression (univariate analysis: HR = 2.83, 95% CI: 1.73-4.65, p < 0.0001, *I^2^* = 57.8%; multivariate analysis: HR = 2.35, 95% CI: 1.42-3.89, p = 0.0009, *I^2^* = 0%)].

The miRNAs that were targeted by these circRNAs were also identified in six of the nine included studies, revealing the mechanisms through which the circRNAs were involved in tumorigenesis and cancer progression. CircPARD3 acts as a sponge of the tumor-suppressor miR-145-5p in LSCC (22), while circCORO1C sponges let-7c-7p whose normal function is to inhibit the proliferation, migration and invasion of LSCC cells and to promote their apoptosis (21). Circ0000199 and circ0072387 interact simultaneously with the tumor suppressor miR-145-5p & miR-29b-3p and hsa-miR-129-3p, hsa-miR-141-3p, & hsa-miR-29-3p respectively in OSCC cells thereby regulating cell proliferation, growth and differentiation (24, 34). In nasopharyngeal carcinoma, circMYC is reported to interact with miR-20b-5p and let-7e-5p simultaneously, which in turn target argonaute RISC component 1 (AGO1) and cryptochrome circadian regulator 2 (CRY2) (42). Both AGO1 and CRY2 tightly regulate cell development, proliferation and migration (49, 50). Finally, circPVT1 binds to miR-497-5p through a specific binding site and affects its expression, leading to downregulation of miR-497-5p in HNSCC tumors with mutant p53 (13). The downregulation of both circ_00072387 and circ_0092125 was reported to significantly affect tumor size, TNM stage, and lymph node metastasis in OSCC patients by interacting with respective miRNAs (33, 34). Circ_00072387 binds with multiple miRNAs such as miR-129-3p, miR-141-3p, and miR-29-3p which regulate several tumor related pathways *viz*; MAPK signaling pathway, Ras signaling pathway, and Hippo signaling pathway (34). Similarly, circ_0092125 interacts with miR_1184, miR_1205, and miR_1322 which are reported to be involved in the occurrence and progression of OSCC (33). In nasopharyngeal carcinoma, interaction of circMYC with miR-20b-5p and let-7e-3p regulates tumor development as both these miRNAs exhibit tumor suppressor functions in a variety of cancers (42). Luo et al., 2020 also reported that miR-20b-5p and let-7e-3p jointly target AGO1 and CRY2. Various reports indicate the crucial role played by AGO1 in solid tumors and the involvement of CRY2 in epithelial-mesenchymal transition, Akt signaling and p53 signaling (42).

In addition, some of the included studies also revealed other molecular mechanisms through which the identified circRNAs contributed towards the cancer phenotype. The upregulation of circCUX1 was associated with radiotherapy resistance of hypopharyngeal tumors attributed to the binding of circCUX1 to the 3’UTR-of caspase-1, thereby inhibiting its expression and resulting in a decrease in inflammatory factors, thus leading to tolerance to radiotherapy (18). Similarly, circPARD3 promoted the proliferation, migration, invasion and chemoresistance of LSCC by inhibiting autophagy *via* the PRKC1-Akt-mTOR pathway (22). CircCORO1C promoted the proliferation, migration and invasion of LSCC cells by specifically upregulating the expression of the target PBX3 gene and affecting the epithelial mesenchymal transition (EMT) process. Indeed, dysregulation of PBX3 has also been reported in prostate, gastric, cervical and liver cancer (21). CircPVT1 was significantly upregulated in tumors with mutant p53 compared to those with wild type p53, and only in HNSCC patients with p53 mutations. In fact, the expression of circPVT1 was found to be regulated at the transcriptional level by the mut-p53/YAP/TEAD complex which resides on the promoter of circPVT1, such that increased expression of mutant p53, YAP and TEAD1 can increase the nascent circPVT1 expression. Simultaneously, circPVT1 might act within a positive self-regulating loop, controlling and enhancing its own nuclear expression, and thus regulating tumorigenesis (13).

Limitations: Our current study has some limitations which should be considered: (a) Based on our inclusion criteria, eight out of nine studies were only from Asian population (b) The sample size and number of the enrolled studies in this analysis were relatively small (c) Studies with good quality diagnostic data could not be retrieved because of which it was not possible to determine the potential of circRNAs as diagnostic markers of HNSCC (d) Begg’s funnel plot and Egger’s test value could not be generated for studies reporting downregulation of circRNAs in HNSCC due to the small number of studies. Thus, more high-quality studies on larger sample sizes and diverse population groups are required in order to firmly establish circRNAs as a good prognostic marker of HNSCC.

## Conclusion

Taken together, our meta-analysis indicates that the nine circRNAs (circPVT1, circCOROC1, circPARD3, circ_0000199, circMYC, circ_0102272, circCUX1, circ_0092125 and circ_00072387) are potential prognostic biomarkers of HNSCC.

## Data Availability Statement

The original contributions presented in the study are included in the article/supplementary material. Further inquiries can be directed to the corresponding author.

## Author Contributions

MN and DR contributed to the design of study, performed the statistical analyses and drafted the manuscript. YC designed the study, provided intellectual input, approved the protocols to be followed in the study and drafted the manuscript. All authors read and approved the final manuscript.

## Conflict of Interest

The authors declare that the research was conducted in the absence of any commercial or financial relationships that could be construed as a potential conflict of interest.

## Publisher’s Note

All claims expressed in this article are solely those of the authors and do not necessarily represent those of their affiliated organizations, or those of the publisher, the editors and the reviewers. Any product that may be evaluated in this article, or claim that may be made by its manufacturer, is not guaranteed or endorsed by the publisher.
